# A case report of rhino-facial mucormycosis in a non-diabetic patient with COVID-19: a systematic review of literature and current update

**DOI:** 10.1186/s12879-021-06625-3

**Published:** 2021-09-03

**Authors:** Faezeh Mohammadi, Milad Badri, Shapoor Safari, Nima Hemmat

**Affiliations:** 1 Department of Medical Parasitology and Mycology, School of Medicine, Qazvin University of Medical Sciences, Shahid Bahonar Blvd, PO Box: 34199-15315, Qazvin, Iran; 2 Medical Microbiology Research Center, Qazvin University of Medical Sciences, Qazvin, Iran; 3 Department of Otolaryngologist, Fellowship of Rhinology, Razi Hospital, Qazvin, Iran; 4 Department of Cellular and Molecular Research Center, Qazvin University of Medical Sciences, Qazvin, Iran; 5 Metabolic Diseases Research Center, Research Institute for Prevention of Non-Communicable Diseases, Qazvin University of Medical Sciences, Qazvin, Iran

**Keywords:** COVID-19, Mucormycosis, *Rhizopus oryzae*

## Abstract

**Background:**

COVID-19 disease may be associated with a wide range of bacterial and fungal infections. We report a patient with COVID-19 infection who developed rhino-facial mucormycosis during treatment with corticosteroids.

**Case presentation:**

A 59-year-old non-diabetic male patient was admitted with a diagnosis of COVID-19 based on positive RT-PCR and CT of the lungs. Due to sever lung involvement, he was treated with methylprednisolone. The patient was re-admitted to hospital, due to nasal obstruction and left side facial and orbital swelling, several days after discharge. In sinus endoscopic surgery, debridement was performed and the specimens were sent to pathology and mycology laboratories. A nasal biopsy showed wide hyphae without septa. The sequenced PCR product revealed *Rhizopus oryzae*. Despite all medical and surgical treatment, the patient died. In addition, the characteristics of patients with COVID-19-associated mucormycosis were reviewed in 44 available literatures. In most studies, diabetes mellitus was the most common predisposing factor for mucormycosis.

**Conclusion:**

Our report highlights the need for assessing the presence of mucormycosis in patients with COVID-19 and also it shows that physicians should consider the potential for secondary invasive fungal infections in COVID-19 cases.

## Background

COVID-19 is a viral disease of the respiratory tract that continues to be a major health issue worldwide. The disease is associated with common symptoms such as fever, dry cough, fatigue, and shortness of breath and sometimes in severe cases, leads to acute respiratory distress syndrome (ARDS) [1]. On the other hand, the use of corticosteroids to modulating lung injury and reduce mortality in COVID-19 patients may be exposes the patient to opportunistic bacterial and fungal infections [2].

Invasive pulmonary aspergillosis is one of the fungal diseases that complicates COVID-19 manifestations [3]. Moreover, mucormycosis as an opportunistic fungal infection can progress rapidly in immunocompromised patients. The most common clinical form of this fungal infection is rhino-cerebral mucormycosis [4]. We reported a case of rhino facial mucormycosis in a 59-year-old non-diabetic male patient with COVID-19 following corticosteroid treatment, which eventually resulted in death.

## Case presentation

**Fig. 1:**
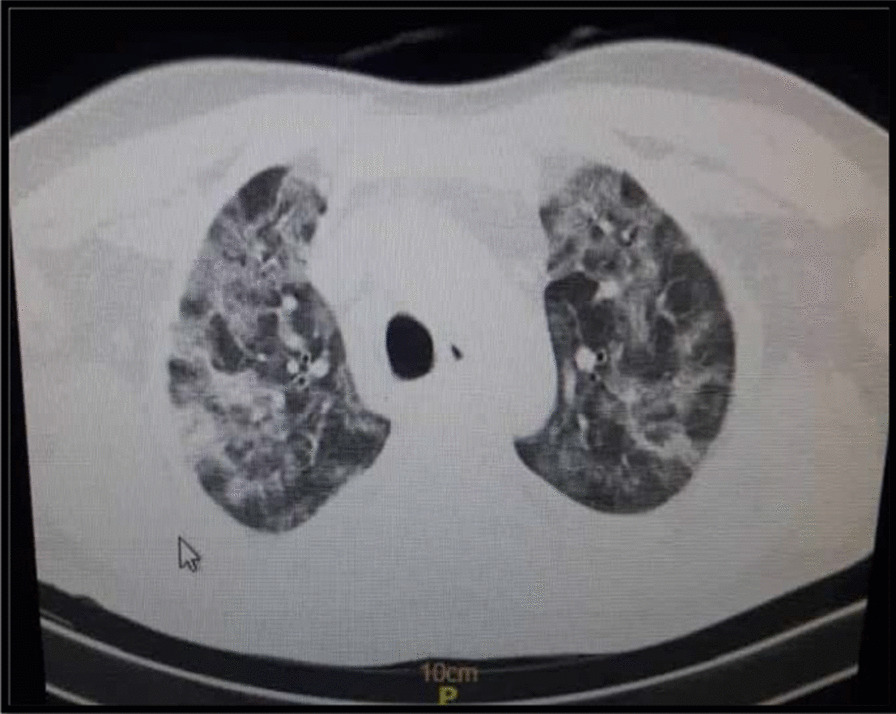
Computed tomography (CT) scan of the chest of a patient with COVID-19 shows multiple patchy ground-glass opacities

A 59-year-old non-diabetic male patient without any underlying disease with clinical symptoms of cough, shortness of breath, and oxygen saturation of 76% was admitted to Razi Hospital, Qazvin, Iran. His vital signs included body temperature of 37.6 °C, blood pressure value of 140/85 (mm Hg) and oxygen saturation of 76%. Positive results of chest X-ray test (CXR), computed tomography (CT) scan of lungs and positive reverse transcriptase polymerase chain reaction (RT-PCR) showed a definite diagnosis of COVID-19 (Fig. 1).

**Fig. 2:**
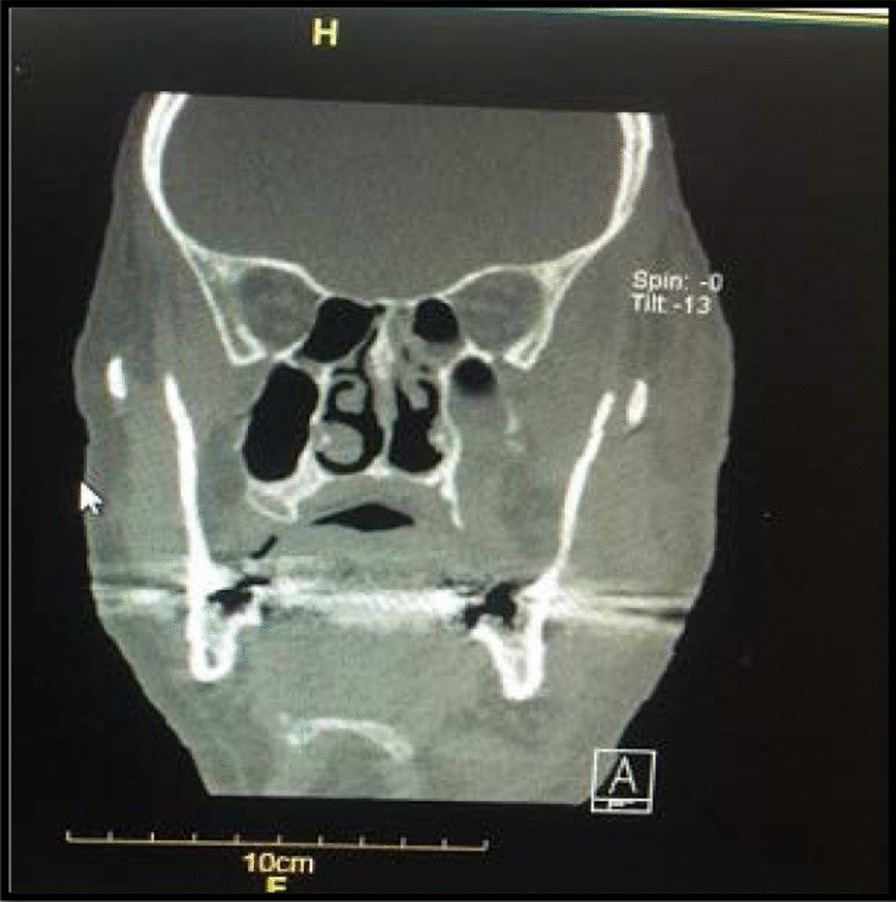
CT scan shows involvement of the left ethmoid, sphenoid, maxillary and paranasal sinuses in a patient with mucormycosis

**Fig. 3:**
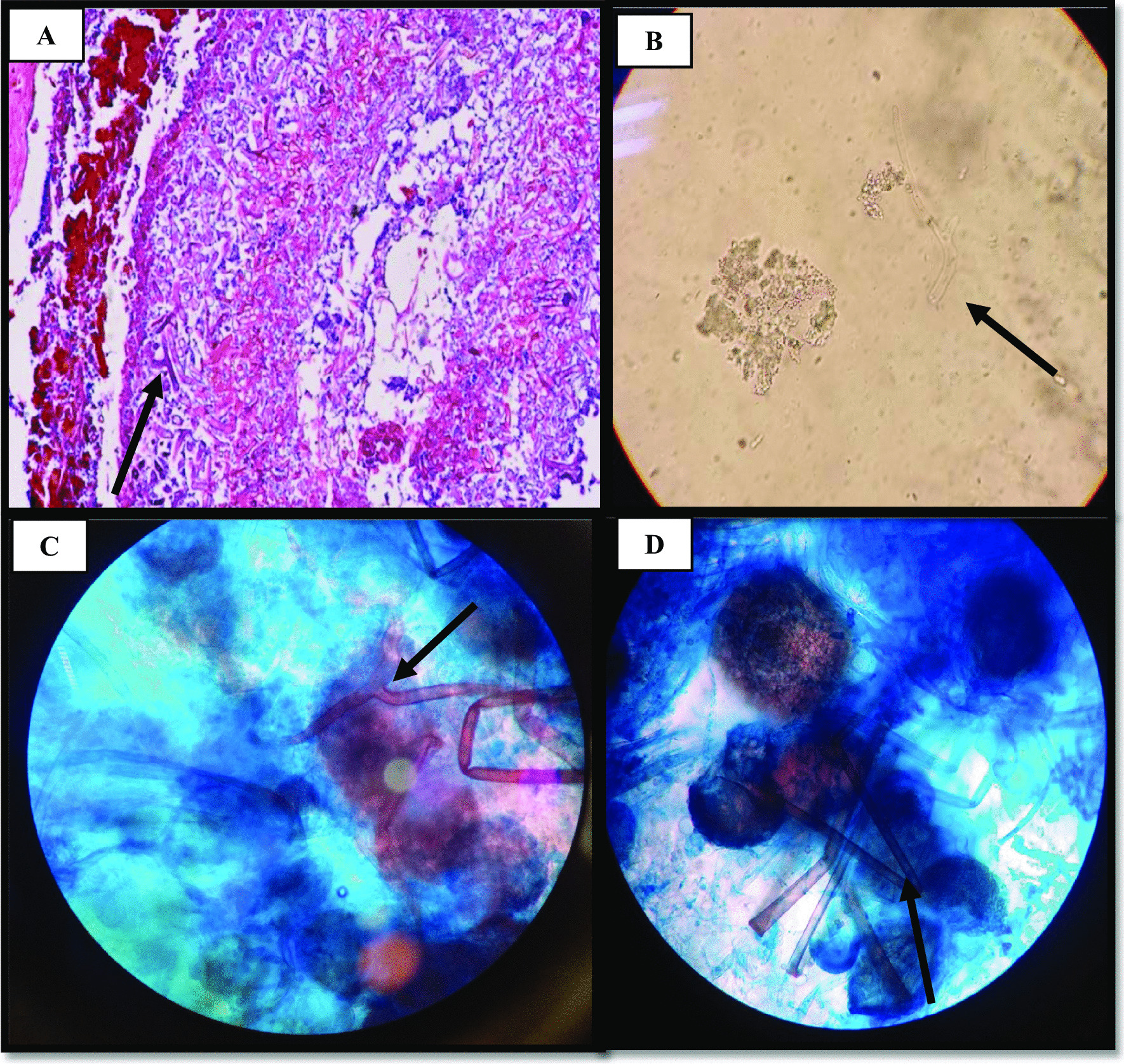
**a** Presence of irregular and non-septate hyphae in H&E staining of pathology. **b** Observation of broad aseptate hyphae in surgical debridement in direct examination (10% KOH). **c** and **d** Lactophenol cotton blue (LCB) mount showed nonseptate hyphae, rhizoids and spore-filled sporangiophores

He was treated with remdesivir injection at a dose of 250 mg stat and then 100 mg daily. The patient was under supportive care for six days, and thereafter methylprednisolone was administered at a dose of 250 mg stat and then 125 mg for 3 days. After 10 days, the patient was discharged while he was relatively in good general condition. Four days after his discharge, the patient was re-admitted to hospital because of nasal obstruction and left side facial and orbital swelling. Table 1 shows the laboratory findings of the patient during both COVID-19 and mucormycosis. Subsequently, the patient visited by an infectious disease specialist and due to the involvement of the left ethmoid, sphenoid, and maxillary sinuses, a CT scan was performed (Fig. 2). In sinus endoscopic surgery, by Rhinologist, severe involvement and necrosis of the left side lateral nasal wall, floor, and septum as well as left ethmoid and sphenoid sinuses were observed and also destruction of the left orbital floor and medial wall were observed. Since clinical results confirmed the possibility of mucormycosis in the patient, treatment with IV liposomal amphotericin B (3 mg/kg/day, according to local guidelines [5]) was started. In addition, the patient underwent daily paranasal sinuses debridement and irrigation with diluted amphotericin B. Biopsy of sinonasal area was made and the specimens were sent to both pathology and mycology laboratories. Examination of the results with haematoxylin and eosin (H&E) staining and direct experiment with 10% potassium hydroxide (KOH) showed irregular hyphae, wide and aseptate (Fig. 3).

**Table 1:** The measured hematological biomarkers in blood of the patient

Categorization of hematological factor	At the time of admission to	
	Covid-19	Mucormycosis
White Blood Cell (WBC) Count, µL	10,400	18,100
Red Blood Cell Count (RBC),million/µL	5.1	4.5
Hemoglobin (Hb), mg/dL	15.8	14.1
Platelets, µL	136,000	137,000
Neutrophil count, **%**	88.1	94.7
Lymphocyte count, %	7.7	1.8
C-Reactive Protein (CRP)	48.1	55.8
Blood urea nitrogen (BUN), mg/dL	25.2	28.4
Serum creatinine, mg/dL	1.59	1.24
Sodium Blood, mEq/L	146	144
Potassium Blood,mEq/L	4	4.6

**Fig. 4:**
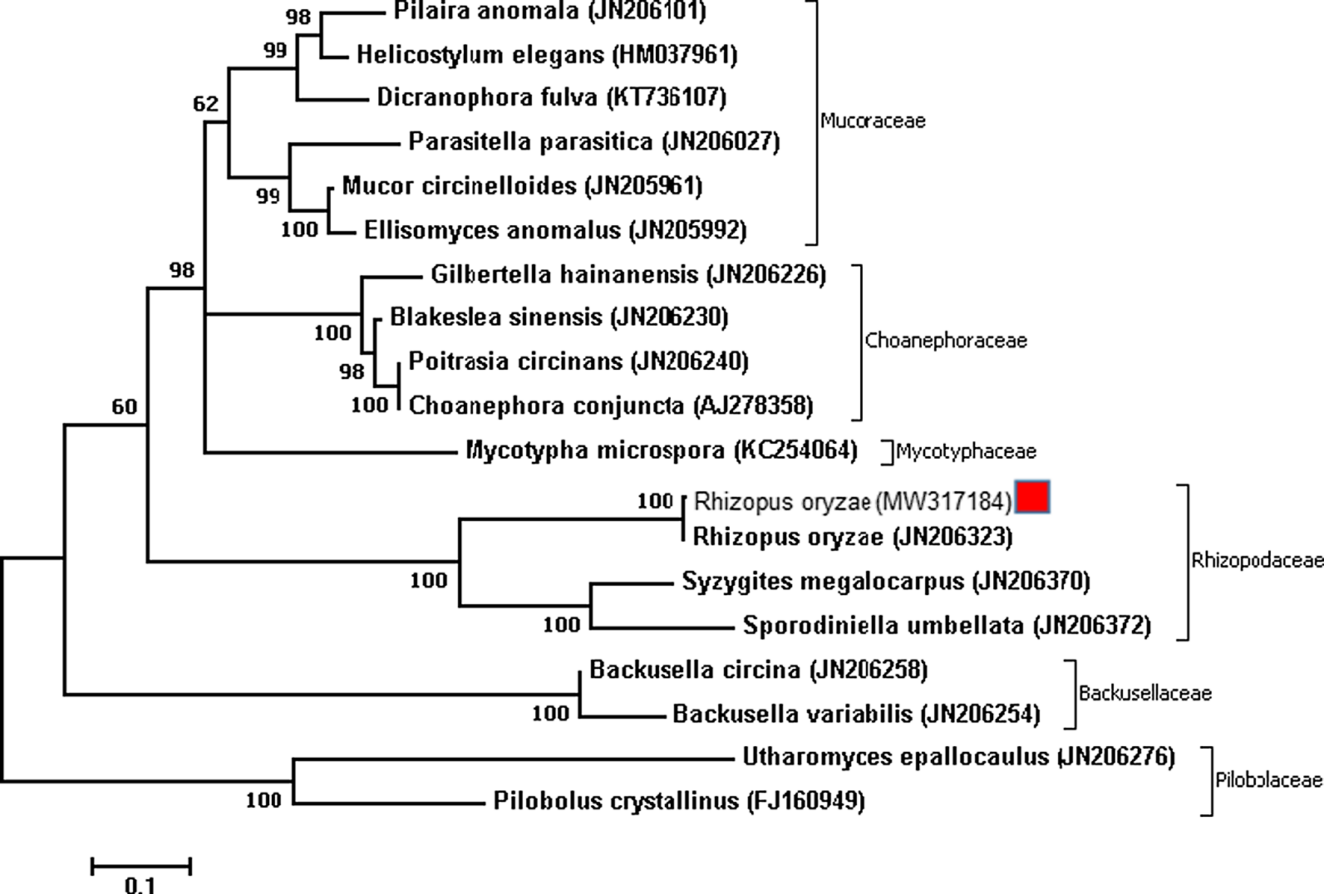
The phylogenetic tree of isolates of *Rhizopus oryzae.* Based on ITS sequence from a patient with mucormycosis and GenBank sequences of some related species were estimated in MEGA7 using the ML analyses based on 1,000 bootstrap replications

In addition, the sample was inoculated on PDA (Sigma-Aldrich,UK) and was incubated for 4–5 days at 37 °C. After colony growth, non- septate hyphae, rhizoids and spore-filled sporangiophores were observed in slides prepared with LCB (Fig. 3). The antifungal susceptibility testing was performed in 96-well plates by following the M38-A2 guidelines of the CLSI for in vitro testing. MIC values against AMB, ITC and VRC were 0.5 mg/mL, 16 mg/mL and 32 mg/mL, respectively. In the next step, DNA extraction was carried out using the glass beads and phenol:chloroform:isoamyl alcohol (25:24:1) method, previously described [6]. The fungal isolate was identified by molecular analysis of ITS1-5.8S-ITS2 region using the primers for ITS1 (5′-TCCGTAGGTGAACCTGCGG-3′) and ITS4 (5′-TCCTCCGCTTATTGATATGC-3′). The sequenced PCR product showed 100% sequence identity with *R. oryzae* and it was registered in the GenBank database under the assigned accession number MW317184. Sequence was aligned with using the ClustalW algorithm as implemented in Bioedit version 7 (http://www.mbio.ncsu.edu/BioEdit/bioedit.html). The molecular diversity of the sample was estimated by phylogenetic analysis via MEGA7 software. In order to compare the sequences with available DNA sequences in GenBank, the nucleotide BLAST with the BLASTn algorithm was applied through CLUSTAL omega (https://www.ebi.ac.uk/Tools/msa/clustalo/). The protocols were conducted based on the ML method using the Tamura-Nei model. The number of bootstrap replications was considered to be 1000 (Fig. 4). Because of the progression of the disease and the involvement of the cheeks and orbit, necrotic tissues were removed. Despite all measures, the patient unfortunately expired on the seventh day of his admission due to loss of consciousness and involvement of central nervous system.

## Discussion and conclusions

COVID-19 disease has a rapid and widespread distribution with mild to severe symptoms. Supportive care, corticosteroids and remedial drugs are good treatment options in COVID-19. On the other hand, due to the use of steroids, these patients may be susceptible to invasive mould infections. Furthermore, diabetes mellitus complicates the management of Covid-19 infection.

Mucormycosis is an acute fungal infection caused by the members of mucoraceae family. Mucormycosis in uncontrolled diabetic patients and immunocompromised is an opportunistic and fatal fungal disease [7]. The most common clinical manifestation of mucormycosis in immunocompromised patients is the rhino-orbito-cerebral form [8]. Infection begins in the nasal cavities and paranasal sinuses. The symptoms of mucormycosis include one-sided facial swelling, headache, fever, inflammation, eyelid drooping and black lesions on nasal that the disease spreads rapidly [9]. Infarction and necrosis of host tissues occur due to invasion of non-septate hyphae [10]. Methods for diagnosing mycromycosis include histopathology, direct testing, and culture of clinical specimens [11]. The first line of management of mucormycosis is recommended injection of liposomal amphotericin B. In case of intolerance of the treatment regimen or general weakness of the patient, azole compounds such as Posaconazole and Isavuconazole can be used [5].

The database search using the terms ‘‘COVID’’ OR ‘‘SARS-CoV2’’ OR ‘‘Coronavirus’’ AND "Mucor" OR ‘‘Zygomycosis’’ revealed a total of 44 articles. The characteristics of patients with COVID-19-associated mucormycosis were shown in Table 2.

**Table 2:** Characteristics of patients with mucormycosis and COVID-19 reported in the literature

Country (case)	Mean age	Sex	Underlying conditions	Type of Mucormycosis	Outcome
India (n = 110)	53.5	M:86,F:24	DM:88,DKA:9 CKD:9, RT:2 SOT:1,CGD:1 DCLD:1,TB:1	Rhino-Facial: Majority Pulmonary:2.7%	Died:27,Alive:74 LFU:4, Unchanged:4 LAMA:5
Iran (n = 20)	51.6	M:11,F:9	DM:15,HM:2	Rhino-Facial	Died:8, Alive:12
Turkey (n = 12)	64.5	M:9,F:3	DM:9	ROM, ROCM	Died:8,Alive:4
Egypt (n = 11)	53	M:7,F:4	DM:7	ROCM	Died:5,Alive:6
USA (n = 10)	51.4	M:8,F:2	DM:7	ROCM:3,ROM:3 Pulmonary:4	Died:8, Alive:2
The Netherlands (n = 4)	60	M:4	DM:2, CLL:1	ROCM, Pulmonary Disseminated	Died:3, Alive:1
UK (n = 2)	22	M:2	Hypothyroidism:1 Obesity:1	Disseminated	Died:2
Spain (n = 2)	55	M:2	DM:1, RT:2	Rhino-Facial, Musculoskeletal	Alive:2
Australia (n = 1)	53	M:1	MDS, AML	Pulmonary	Died
Brazil (n = 1)	86	M:1	HTN	GIM	Died
France (n = 1)	55	M:1	FL, HCT	Pulmonary	Died
Mexico (n = 1)	24	F:1	DKA	ROM	Died
Italy (n = 1)	66	M:1	HTN	Pulmonary	Died
Iraq (n = 1)	53	M:1	DM	ROCM	Died

*F* female; *M* male; *DM* diabetes mellitus; *HTN* hypertension; *CKD* chronic kidney disease; *SOT* solid organ transplant; *DCL*D Decompensated chronic liver disease; *AML* Acute myeloid leukemia; *MDS* Myelodysplastic syndrome; *DKA* Diabetic ketoacidosis; *RT* Renal transplant; *FL* Follicular lymphoma; *HCT* Hematopoietic cell transplantation; *TB* Tuberculosis; *ROM* rhino-orbital mucormycosis; *ROCM* rhino-orbito-cerebral mucormycosis; *GIM* gastrointestinal mucormycosis

Review of literature published till June 2021 shows that the most cases are related to mucormycosis in COVID-19 patients was in India with 110 cases [12–25], followed by Iran (20 cases) [26–30], Turkey (12 cases) [31, 32], Egypt (11 cases) [33, 34], the United States and (10 cases) [35–43], the Netherlands (4 cases) [44], UK and Spain (2 cases) [45–47]. Furthermore, a case of mucormycosis in COVID-19 patients has been published from Brazil [48], Australia [49], France [50], Mexico [51], Italy [52] and Iraq [53]. Studies show that the median age of the patients was 53.4 years (range 22–86) with a higher prevalence of mucormycosis in men (75.7%). The association of mucormycosis with uncontrollable diabetes has been proven [54]. Diabetes mellitus was the most common predisposing factor (73.4%) for mucormycosis in COVID-19 patients [14, 15, 27, 32, 35, 44]. In 5 cases (2.8%), no risk factors for mucormycosis were reported [14, 21, 29, 37]. In our reported case, corticosteroid-related hyperglycemia was observed in a patient with no history of diabetes. Predisposing factors for mucormycosis include diabetes mellitus, neutropenia, corticosteroid use, and immunodeficiency, among which diabetes is the most common risk factor linked with mucormycosis [55]. The severity of COVID-19 infection and its dangerous consequences are higher in individuals with diabetes. Glucocorticoids reduce mortality in patients with COVID-19 by reducing cytokine storm. Nevertheless, corticosteroids can increase the risk of fungal and bacterial secondary infections [56]. Therefore, use of steroids should be avoided in mild to moderate COVID-19 cases as they lead to dangerous results. Reports indicate that 82% of patients received corticosteroids.

The mean duration from between diagnosis of COVID-19 and the onset of symptoms of mucormycosis was 15 days [15, 18]. Studies show that the most common clinical manifestation of mucormycosis is rhino-facial, followed by pulmonary and disseminated form. Herein, we report a case of mucormycosis in a 59-year-old male non-diabetic with COVID-19. The patient developed rhino-facial mucormycosis after the initiation of corticosteroid. The mean duration between diagnosis of COVID-19 infection and the onset of symptoms of mucormycosis was 15 days [13, 15, 57]. The present case indicates that in the presence of COVID-19, even short-term treatment with corticosteroids may be a predisposing factor in leading the patient to rhino-orbital mucormycosis. Studies show that glucose control, timely treatment with liposomal amphotericin B, and surgical debridement are effective in the management of mucormycosis. The prognosis of the disease depends on factors such as early diagnosis and management to limit the spread of infection into the intracranial space [5, 58]. This study, in line with the results of other studies, reveals that the possible occurrence of secondary invasive fungal infections in patients with COVID-19 infection should not be neglected. Effort to maintain blood sugar and the rational use of corticosteroids in COVID-19 patients is recommended to reduce the risk of mucormycosis.

## Data Availability

All data analyzed during this study are included in this published article.

## Abbreviations

COVID-19: Coronavirus disease 2019
ARDS: Acute respiratory distress syndrome
H&E: Hematoxylin and eosin
PDA: Potato dextrose agar
CLSI: Clinical and Laboratory Standards Institute
MIC: Minimum inhibitory concentration
*R.** oryzae*: *Rhizopus oryzae*
ML: Maximum-likelihood
